# Outdoor malaria transmission in forested villages of Cambodia

**DOI:** 10.1186/1475-2875-12-329

**Published:** 2013-09-17

**Authors:** Lies Durnez, Sokny Mao, Leen Denis, Patricia Roelants, Tho Sochantha, Marc Coosemans

**Affiliations:** 1 Department of Biomedical Sciences, Institute of Tropical Medicine, Nationalestraat 155, Antwerpen B-2000, Belgium; 2 National Center for Malaria Control, Parasitology and Entomology, Phnom Penh, Cambodia; 3 Department of Biomedical Sciences, University of Antwerp, Antwerp, Belgium

## Abstract

**Background:**

Despite progress in malaria control, malaria remains an important public health concern in Cambodia, mostly linked to forested areas. Large-scale vector control interventions in Cambodia are based on the free distribution of long-lasting insecticidal nets (LLINs), targeting indoor- and late-biting malaria vectors only. The present study evaluated the vector density, early biting activity and malaria transmission of outdoor-biting malaria vectors in two forested regions in Cambodia.

**Methods:**

In 2005 two entomological surveys were conducted in 12 villages and their related forest plots in the east and west of Cambodia. Mosquitoes were collected outdoors by human landing collections and subjected to enzyme-linked immunosorbent assay (ELISA) to detect *Plasmodium* sporozoites after morphological identification. Blood samples were collected in the same villages for serological analyses. Collected data were analysed by the classification and regression tree (CART) method and linear regression analysis.

**Results:**

A total of 11,826 anophelines were recorded landing in 787 man-night collections. The majority (82.9%) were the known primary and secondary vectors. Most of the variability in vector densities and early biting rates was explained by geographical factors, mainly at village level. Vector densities were similar between forest and village sites. Based on ELISA results, 29% out of 17 *Plasmodium*-positive bites occurred before sleeping time, and 65% in the forest plots. The entomological inoculation rates of survey 1 were important predictors of the respective seroconversion rates in survey 2, whereas the mosquito densities were not.

**Discussion:**

In Cambodia, outdoor malaria transmission in villages and forest plots is important. In this context, deforestation might result in lower densities of the primary vectors, but also in higher densities of secondary vectors invading deforested areas. Moreover, higher accessibility of the forest could result in a higher man-vector contact. Therefore, additional vector control measures should be developed to target outdoor- and early-biting vectors.

## Background

Within the Greater Mekong Region, progress in malaria control has been substantial over the last ten years. However malaria remains an important public health concern in some provinces of each country [1]. In 2010, Cambodia reported 49,356 confirmed malaria cases [2]. It is estimated that 2.1 million people (15% of the population) in Cambodia are at risk of malaria, of which approximately half a million live in forest and forest-fringe areas with high malaria transmission [3]. Despite active and uncontrolled deforestation, about 61% of the total Cambodian land area was estimated to be covered with forest in 2002 [4], of which more than 80% is located in malaria-endemic areas [5]. Most of these forested areas are located in provinces bordering Vietnam, Laos and Thailand. People living in villages at the edge of the forest or having forest activities are at high risk of malaria because of the presence of the highly efficient forest malaria vectors *Anopheles dirus s*.*s*. and *Anopheles minimus s*.*s*. [6-9]. Because of the complexity of interactions that may involve vector populations in and outside the forest, it is not easy to predict the impact of deforestation on malaria transmission in this context [5].

Large-scale vector control interventions occurred during the last years in Cambodia, particularly based on the free distribution of long-lasting insecticidal nets (LLINs) [2]. This has contributed to a substantial decrease in malaria cases. However these LLINs only protect people when they are sleeping inside the houses. The main vectors *An*. *dirus s*.*s*. and *An*. *minimus s*.*s*. are exophagic and exophilic, jeopardizing the impact of LLINs [10]. Several studies in Vietnam, and Thailand show that *Anopheles dirus s*.*l*. and *Anopheles minimus s*.*l*. are outdoor and early biters [11-13]. In Vietnam, a higher vector abundance and malaria transmission was observed in forest camps as compared to the nearby village, and a higher risk was observed of being bitten by a *Plasmodium*-infected mosquito during the early evening as compared to the rest of the night [11]. Also, in western Cambodia, this phenomenon of early and outdoor biting has been reported [14], meaning that additional vector control measures are necessary. Additionally, human risk behaviour favouring exposure to malaria vectors, ie staying outside during the night, presents a great challenge. Besides the major forest vectors *An*. *dirus s*.*s*. and *An*. *minimus s*.*s*., a large number of other anopheline species occur in the vicinity of human dwellings. Transmission by ‘secondary’ vectors that have outdoor or early biting behaviour might become more important than transmission by primary vectors in the context of high coverage of insecticide-treated nets (ITNs) [15]. As secondary vectors are often less anthropophilic, and might be more exophagic and early biting, the planning of vector control should take into account their behaviour. Moreover, as pointed out in [11], secondary vectors might be better vectors of *Plasmodium vivax* as compared to *Plasmodium falciparum*, as the extrinsic incubation period of *P*. *vivax* is shorter. In Vietnam, *Anopheles sawadwongporni*, a very early biting secondary vector, was found positive for *P*. *vivax*[11].

Recently, Cambodia has declared its intention to eliminate malaria by 2025 [16]. It is therefore important to study to which extent the malaria vectors in Cambodia are outdoor-biting (exophagic) and early biting, and to assess the importance of secondary vectors in this context. However, facing the decrease of malaria transmission as a result of the control programme, entomological surveys are not sensitive enough to estimate changes in transmission intensity. In this context, serology is being proposed as an additional tool, as proxy for malaria transmission [17] for measuring the force of malaria infection [18].

In the framework of a larger study on the force of malaria infection in the forested environment in Cambodia the results of an entomological survey designed to have a better understanding of early and outdoor malaria transmission by primary and secondary vectors in forested areas in the eastern and western part of Cambodia, are presented here. Vector abundance, malaria transmission, and early biting rates in forest camps were compared to the situation in the nearby village. Results obtained from the entomological survey are analysed in relation to the serological data obtained in the same localities during the same time frame [19]. The study was performed in 2005 and will be used as a reference to assess the entomological situation in a fast-changing environment.

## Methods

### Study sites

**Figure 1:**
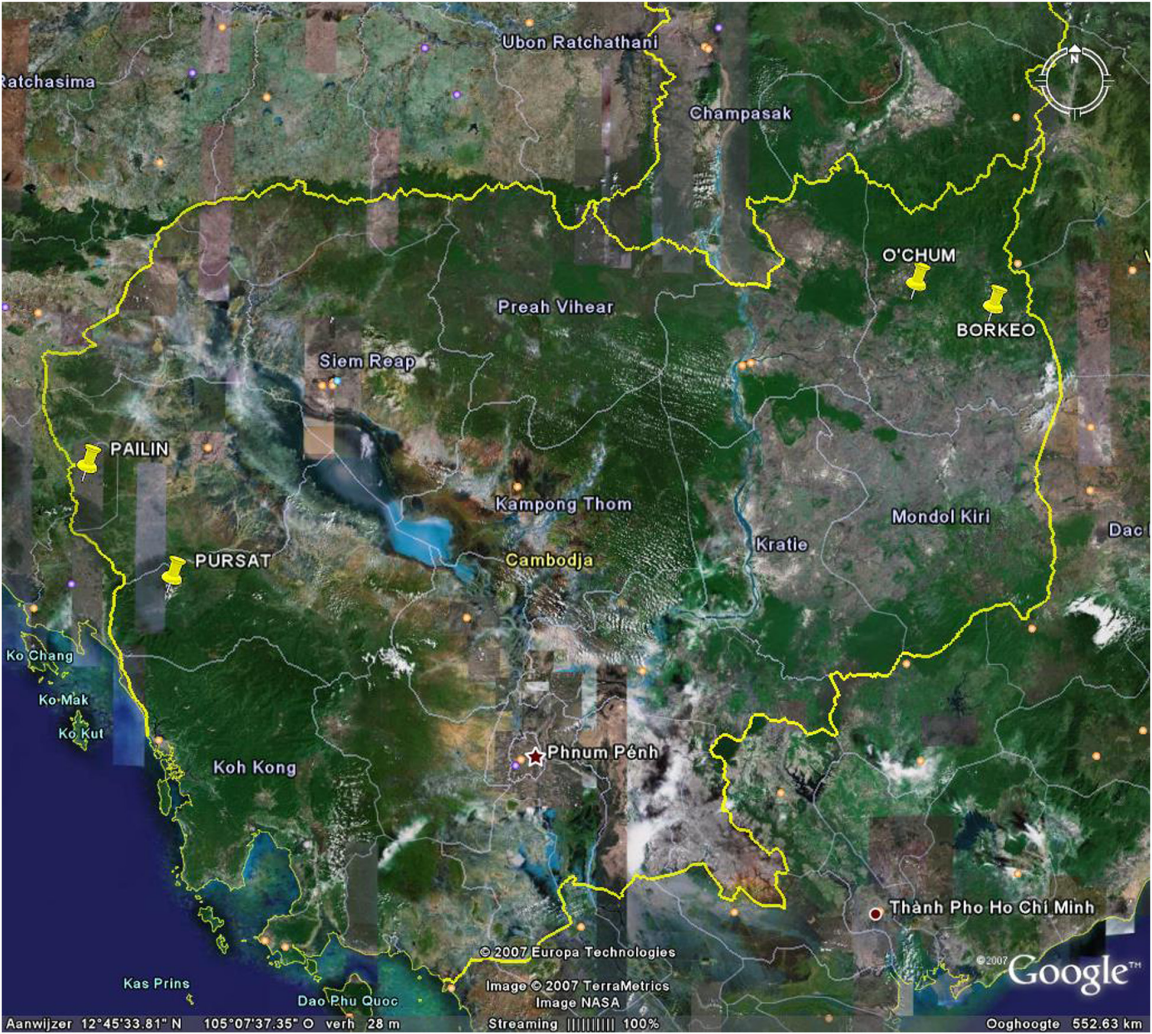
**Overview of Cambodia with the four districts indicated.** Pailin and Pursat are located in the west, O’chum and Borkeo are located in the north-east. (Google Maps).

The study sites were located over five districts in forested areas of Cambodia: two in the north-east (O’Chum and Borkeo in Rattanakiri Province) and three in the north-west (Veal Vang in Pursat Province and Mittapheap/Salakrao in Pailin Province). In each of the districts (Mittapheap and Salakrao were taken together) three villages were selected (Figure 1). The dominant ethnic group in Pailin and Pursat is Khmer, whereas Charay and Tumpurn are dominant in the Rattanakiri villages. In both regions the majority of the inhabitants are engaged in forest-related work activities (agriculture, logging, hunting) and may spend the night in their forest plots during the harvest period. In all villages, domestic animals (including cattle and dogs) are present and roaming freely. The dry season typically runs from November to May and the rainy season from June to October in both regions. The two main malaria vector species are *An*. *minimus s*.*s*. and *An*. *dirus s*.*s*. [7]. A more detailed description of the study sites is given in Table 1.

**Table 1:** Description and location of the villages

**Province**	**Village code**	**District**	**Village**	**Lat**	**Long**	**Inhabitants**	**Type of vegetation**
**Rattanakiri**	**BX**	Borkeo	Sala	13,73164	107,2413	244	Deforested environment
	**BY**	Borkeo	Leutouch	13,7139	107,2465	172	70% evergreen forest
	**BZ**	Borkeo	Saleo	13,775	107,2255	80	Evergreen forest fields
**Rattanakiri**	**OX**	O Chum	Bornhuk 2	13,77195	107,1374	215	Rubber plantation
	**OY**	O Chum	Ping	13,82496	107,0954	136	Scattered forest
	**OZ**	O Chum	Prac	13,82523	107,0538	200	Evergreen forest
**Pursat**	**VX**	Veal Veng	Tang Yo	12,38503	103,2772	250	Evergreen/deciduous/deforested
	**VY**	Veal Veng	Don Neak	12,36678	103,2242	220	Deciduous/deforested
	**VZ**	Veal Veng	Dey Krahorm Leu	12,27406	102,9521	150	Evergreen/deforested
**Pailin**	**PX**	Mittapheap	O-Kting	12,77772	102,7014	300	Evergreen/deforested
	**PY**	Mittapheap	Pang Rolim	12,788	102,6911	200	Deciduous/deforested
	**PZ**	Salakrao	Tick Cheng	12,92217	102,6785	200	Evergreen/deforested

**Figure 2:**
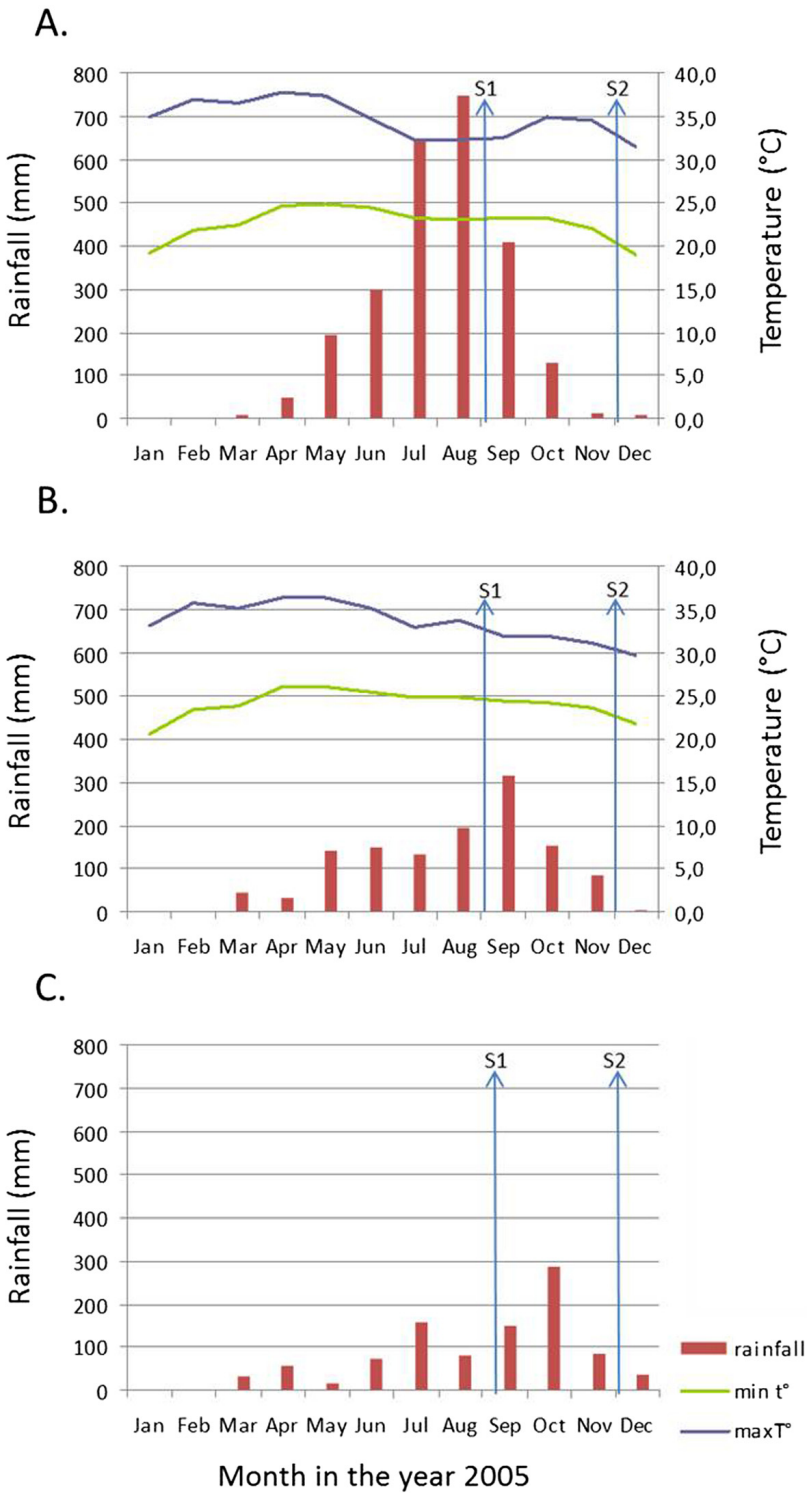
**Meteorological data in Ratanakkiri province (A), Pursat province (B) and Pailin province (C) in 2005.** This includes the monthly rainfall in mm (left axis) and the minimum and maximum temperature in degrees Celsius (right axis). The timing of survey 1 (S1) and survey 2 (S2) are indicated by arrows.

In 2005 the meteorological data (Figure 2) were recorded for the stations of Rattanakiri (Long:106°59', Lati:13°44', Altitude: 330 m), Pursat (Long.103°51', Latitude:12°33', Altitude: 18 m) and Pailin (Long:102°36', Lat:12°48', Altitude: 170 m).

### Census

At the beginning of the study a census was carried out of each village to collect basic information on sleeping habits, education level and net use.

### Mosquito collections

Two entomological surveys (August-September and November-December 2005) were conducted in the 12 forest villages. For each entomological survey, outdoor human landing collections were carried out for six successive nights. Human landing collections lasted from 18.00 until 06.00. Each collector collected mosquitoes for four hours per night. A rotation of collectors was conducted every two days for the different collection points and collection hours. In the village, at the edge, two collection points were selected in the front of two houses, making a collection effort of 12 man-nights per survey. In the forest, two collection points were chosen in forest camps where people of the village have their forest plot or stay temporarily for hunting or logging. The collection effort was 12 to 14 man-nights per survey (in some plots collections were carried out during seven successive nights instead of six). The forest plots were between 0.1 and 4.5 km from the village. The same collection points were maintained throughout the study. For one site, mosquitoes were collected only in the forest camp where people lived permanently (BZ).

Two additional collection sites per village were selected on the way from the village to the forest camp (between 0.5 and 1 km from the village edge). In these sites mosquitoes were collected in the evening (from 18.00 to 22.00) and in the early morning (from 04.00 to 08.00).

Mosquitoes were stored by collection hour and morphologically identified in the field by use of a standardized key for medically important anophelines of Southeast Asia [20]. Mosquitoes were individually stored in small tubes over silica gel for subsequent analysis.

### Laboratory analysis on mosquitoes

Most of the collected mosquitoes were subjected to enzyme-linked immunosorbent assay (ELISA) to detect *P*. *falciparum*, *P*. *vivax 210* and *P*. *vivax 247* circumsporozoite proteins (CSP) in the head-thoracic portion of individual mosquitoes. Details of this procedure and the numbers of mosquitoes tested were published earlier [21]. As false-positive CSP-ELISA occurs in this area, positive CSP ELISA mosquitoes were confirmed by PCR as previously described [21].

The morphological identification of the mosquitoes found positive for ELISA was confirmed by PCR using the PCR-RFLP for *An*. *minimus* complex [22], and the allele specific PCR for *An*. *dirus* complex [23]. The identification of *Anopheles barbirostris s*.*s*. was confirmed by sequencing (GenoScreen, Lille, France) the ITS2 rDNA region using primers ITS2A and ITS2B as described in [24]. The sequences were blasted and compared with reference sequences described in [25]. In addition, molecular identification was obtained for 440 randomly chosen specimens of the *An*. *dirus* complex and 351 randomly chosen specimens of the *An*. *minimus* complex as described above. A random sample of the mosquitoes morphologically identified as *An*. *maculatus* (535 specimens) were identified using a PCR-RFLP, based on the amplification of ITS2 rDNA region using primers ITS2A and ITS2B as described in [24] followed by a restriction using the HaeIII restriction enzyme.

### Collection and analysis of blood samples

Collection of blood samples was carried out as described in [19]. In short, finger-prick blood samples were taken from each member of the household. Microscopy was carried out on all samples to estimate *P*. *falciparum* and *P*. *vivax* parasite rate (PR). The samples were stored desiccated at 4°C. *Plasmodium falciparum* GLURP antibodies and *P*. *vivax* MSP-1_19_ antibodies were detected using ELISA. ELISA optical densities were converted to percentage positivity. A mixture model was used to generate a cut-off for positivity. Seroconversion rates (SCR) were estimated by using a simple reversible catalytic conversion model to fit the dichotomised serological results, using maximum likelihood methods [19].

### Ethical approval

The study was approved by the ethical committees of the National Centre of Malariology CNM in Phnom Penh (Cambodia) and of the Institute of Tropical Medicine of Antwerp (Belgium). The mosquito collectors and householders were informed about the objectives, process and procedures of the study and oral informed consent was sought from them. Collector candidates were invited among the adult village population and if individuals wanted to withdraw they were allowed to do so at any time without prejudice. Access to malaria diagnosis and treatment was guaranteed throughout the study. Informed consent was received from all people who agreed to give blood samples after being given information about the objectives, process and procedures of the study.

### Statistical analysis

The non-parametric classification and regression tree (CART) models (described and used in [19,26]) were used to explore the interactions between the mosquito density, expressed as man biting rate (MBR) - the number of bites per man per night at one collection point-, or early biting rate - early man biting proportion (EBP), the percentage of vectors biting before 22.00- and its discriminants. The analysis was performed using a commercial CART software (Salford Systems Inc, Version 6.6, CA, USA). The settings were as described in [26]. In short, a ten-fold cross-validation was used as estimation method, the Gini criterium and the interclass variance were used as a measure of ‘purity’ of the terminal nodes and the one standard error rule was applied to select the best tree. A minimum terminal node size of 20 was selected to avoid too many splits with few observations. CART also provides a ranking based on the overall contribution of each variable in the construction of the tree. This ranking indicates the relative importance (RI) of each independent variable as a predictor. It is possible that a variable does not occur in the tree but still is ranked as an important predictor because it is identified as the second most important splitter in many nodes [26].

To identify entomological predictors for the epidemiological outcome, linear regression analysis was carried out using STATA 12.0 (Stata Corp. College Station, TX, USA). The dependent variables for which the analyses were carried out separately were the seroconversion rate for *P*. *falciparum* and *P*. *vivax*, and the parasite rate for *P*. *falciparum* and *P*. *vivax*. The dependent variables were transformed first using an arcsine transformation of the square root. The independent variables were the MBR expressed by the number of bites/man/night (B/M/N) of the different vector species of survey 1 and survey 2, the EBP calculated as the percentage of vectors biting before 22.00 of the different vector species of survey 1 and survey 2, and the entomological inoculation rate (EIR) for the respective parasite (*P*. *falciparum* or *P*. *vivax*). Because of the high number of variables, univariate analyses were used to determine the significant variables. Variables with a P-value ≤0.10 were incorporated in a multivariate model. The final linear regression model was obtained by backward selection using a P-value ≤ 0.05 as the criterion and was checked for multicollinearity (by using the vif command in STATA 12.0).

## Results

### Census

A summary of the census results of importance for the interpretation of the presented data is available in Table 2. More details are available in [19].

**Table 2:** Risk factors as obtained by the census

	**East**		**West**	
	**Borkeo**	**O’Chum**	**Pailin**	**Pursat**
N	625	758	778	760
No education (%)	84	71	50	39
Sleeping unprotected in the village (%)	60	30	45	35
Sleeping in the forest (%)	55	34	19	32
Sleeping unprotected in the forest (%)	31	27	17	29

All percentages are related to *N* number of inhabitants.

### Entomological surveys

In a total of 787 man-night collections (of which 295 in the forest camps, 262 in the villages and 230 on the way from the villages to the forest camps, the latter only part of the night), 11,826 anophelines were recorded landing, of which 52.8% were collected in the forest camps, 46.3% in the villages and 1% on the way. The majority of the anophelines (82.9%) were morphologically identified as the known [21] primary and secondary malaria vectors in Cambodia: *Anopheles maculatus sensu lato* (33.1%), *An*. *minimus s*.*l*. (24.8%), *An*. *barbirostris s*.*l*. (14.7%), and *An*. *dirus s*.*l*. (10.,3%). Other anopheline species collected (17.1%) were morphologically identified as *Anopheles philippinensis*, *Anopheles jamesii*, *Anopheles hyrcanus*, *Anopheles karwari*, *Anopheles tessellatus*, *Ano-pheles umbrosis*, *Anopheles kochi*, *Anopheles culicifacies s*.*l*., *Anopheles vagus*, *Anopheles aconitus*, *Anopheles annan-dalei*, and *Anopheles willmori*.

Of the 440 *An*. *dirus* complex members analysed, 99% were molecularly confirmed as *An*. *dirus s*.*s*.. Table 3 shows the molecular identification of the *An*. *minimus* complex and the *An*. *maculatus* complex. The majority of *An*. *minimus* complex members collected in the west were molecularly identified as *An*. *minimus s*.*s*., the specimens morphologically identified as belonging to the *An*. *minimus* complex in the east comprised mainly of *An*. *aconitus*, mixed with *An*. *minimus* s.s. and few other species. The specimens morphologically identified as belonging to the *An*. *maculatus* complex, comprised in the west mainly of *An*. *sawadwongporni* and in the east mainly of a mix of *An*. *maculatus s*.*s*. and *An*. *sawad-wongporni*. As not all specimens collected could be molecularly identified, further analysis will be presented at complex level, based on the morphological identification. Note that in the following, the term *An*. *minimus s*.*l*./*An*. *aconitus* will be used to account for the mix of species in the mosquitoes that were morphologically identified as *An*. *minimus s.l.*

**Table 3:** **Molecular identification of anophelines morphologically identified as *An. minimus s.l. *and *An. maculatus s.l. *in the eastern and the western region of Cambodia, based on PCR-RFLP**

**Morphological identification**	**Molecular identification**	**Group**	**Number identified**	
			**Eastern region**	**Western region**
*An*. *minimus s*.*l*.	*An*. *minimus s*.*s*.	Funestus	16	227
	*An*. *harrisoni*	Funestus	2	
	*An*. *aconitus*	Funestus	75	1
	*An*. *varuna*	Funestus	8	6
	*An*. *pampanai*	Funestus	3	
	*An*. *maculatus s*.*s*.	Maculatus	4	7
	*An*. *vagus*	Subpictus	2	
*An*. *maculatus s*.*l*.	*An*. *maculatus s*.*s*.	Maculatus	134	29
	*An*. *sawadwongporni*	Maculatus	80	279
	*An*. *jamesii*	Jamesii	11	2

In general, mosquito densities per vector complex were similar between forest and village sites. On the way to the forest camps only 115 anophelines were collected, and only during survey 2 in village PY (three bites/night) and in village PZ (6.1 bite/night) of which 77% in the evening and 23% in the early morning. Most of them were *An*. *maculatus s*.*l*. (74.8%), and *An*. *minimus s*.*l*./*An*. *aconitus* (19.1%). *Anopheles dirus s*.*l*. represented 5.2%. Because of these low numbers of specimens collected on the way, further analysis will only focus on the anophelines collected in the forest camps and the villages. Further analyses will be concentrated on the four known malaria vector complexes that were most abundant in this study.

### Biting densities

**Figure 3:**
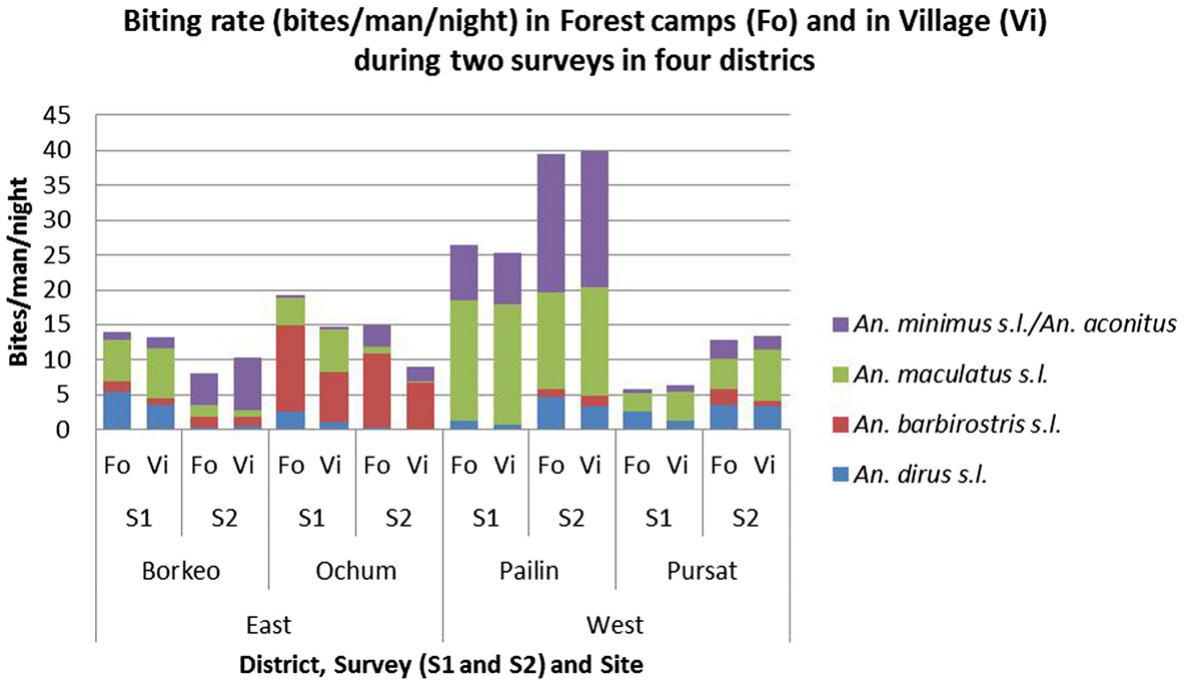
**Average man biting rate of the most abundant anopheline species in the different districts.** Surveys (S1 = survey 1, S2 = survey 2) and collection sites (Fo = forest, Vi = village).

Man-biting rates per district, survey and collection site show a high variability between the districts (Figure 3). The highest densities of *An*. *minimus s*.*l*./*An*. *aconitus* were observed in the district of Pailin and Borkeo. Highest densities of *An*. *barbirostris s*.*l*. were observed in O’chum. Densities of each vector complex were similar between village and the corresponding forest sites.

Analysis by CART (Table 4) shows that for all four vector complexes, the most important determinant of mosquito density was the village (RI of 100 in all species), meaning that most variability is seen between villages. The second most important determinant was the district (RI ranging from 48 to 89; Table 4). The time period (survey) of the collection affected the densities of *An*. *dirus s*.*l*. (RI: 29) and *An*. *minimus s*.*l*./*An*. *aconitus* (RI: 33) but not *An*. *maculatus s*.*l*. (RI: 2) and *An*.*barbirostris s*.*l*. (RI: 0) and this differently according to the district. The relative importance of the sites of collection (forest plot, village) was limited (RI ranging from 0 to 8). All CART trees are shown in Additional file 1.

**Table 4:** **Ranking of predictor variables for *Anopheles *density by their relative importance (RI) as discriminant**

		** RI per mosquito species complex**		
**Discriminants**	***An. dirus s.l.***	***An***. ***minimus s. l. / An. aconitus***	***An. maculatus s.l.***	***An. barbirostris s.l.***
Village	100.00	100.00	100.00	100.00
District	48.33	62.61	78.07	89.40
Region (east/west)	43.77	0.00	2.81	37.74
Survey	29.43	33.16	1.81	0.00
Site (forest/village)	4.83	0.00	0.67	8.00

For *An*. *dirus s*.*l*., besides village (RI: 100) and district (RI: 48), region (RI: 43) and survey (RI: 29) were also important predictors. Only in the villages with highest densities (with on average 3.4 B/M/N compared to 0.435 B/M/N in low density villages), a difference was observed between surveys, which was not consistent for all villages: in some villages (PZ, VZ, both located in the western region), higher densities were observed in the second survey, whereas in other villages (BY, BZ, OZ, PY, VY), higher densities were observed in the first survey. The highest *An*. *dirus s*.*l*. density was observed in the village site of VZ in survey 2 (10.4 B/M/N).

For *An*. *minimus s*.*l*./*An*. *aconitus*, CART shows that the highest densities were observed in the district of Pailin (PY, PZ) and Borkeo (BY, BZ), with a maximum of 28 B/M/N in the forest camps of PY in survey 2. In those villages with high densities, the density was higher in the second survey as compared to the first survey.

For *An*. *maculatus s*.*l*. highest densities were observed in Pailin (PY and PZ), with a maximum of 24 B/M/N in the village site of PY in survey 1.

The highest densities of *An*. *barbirostris s*.*l*. were observed in all three villages of the O’Chum district, with a maximum of 21 B/M/N in the forest site of OZ in survey 1. In the O’Chum district, a higher density was observed in the forest sites as compared to the village sites.

Other less abundant species present in all districts were *An*. *philippinensis*, *An*. *jamesii*, *An karwari*, *An*. *tessellatus*, *An*. *kochi*. Species occasionally found are *An*. *hyrcanus* (BX, BY, BZ,OY, OZ), *An*. *umbrosus* (BZ, OZ, PX, PY, VX), *An*. *culicifacies s*.*l*. (PY, PZ, VY, VZ), *An*. *vagus* (BY, PY, VX and VZ), *An*. *annandalei* (PZ), and *An*. *willmori* (VY).

### Early biting activity

**Figure 4:**
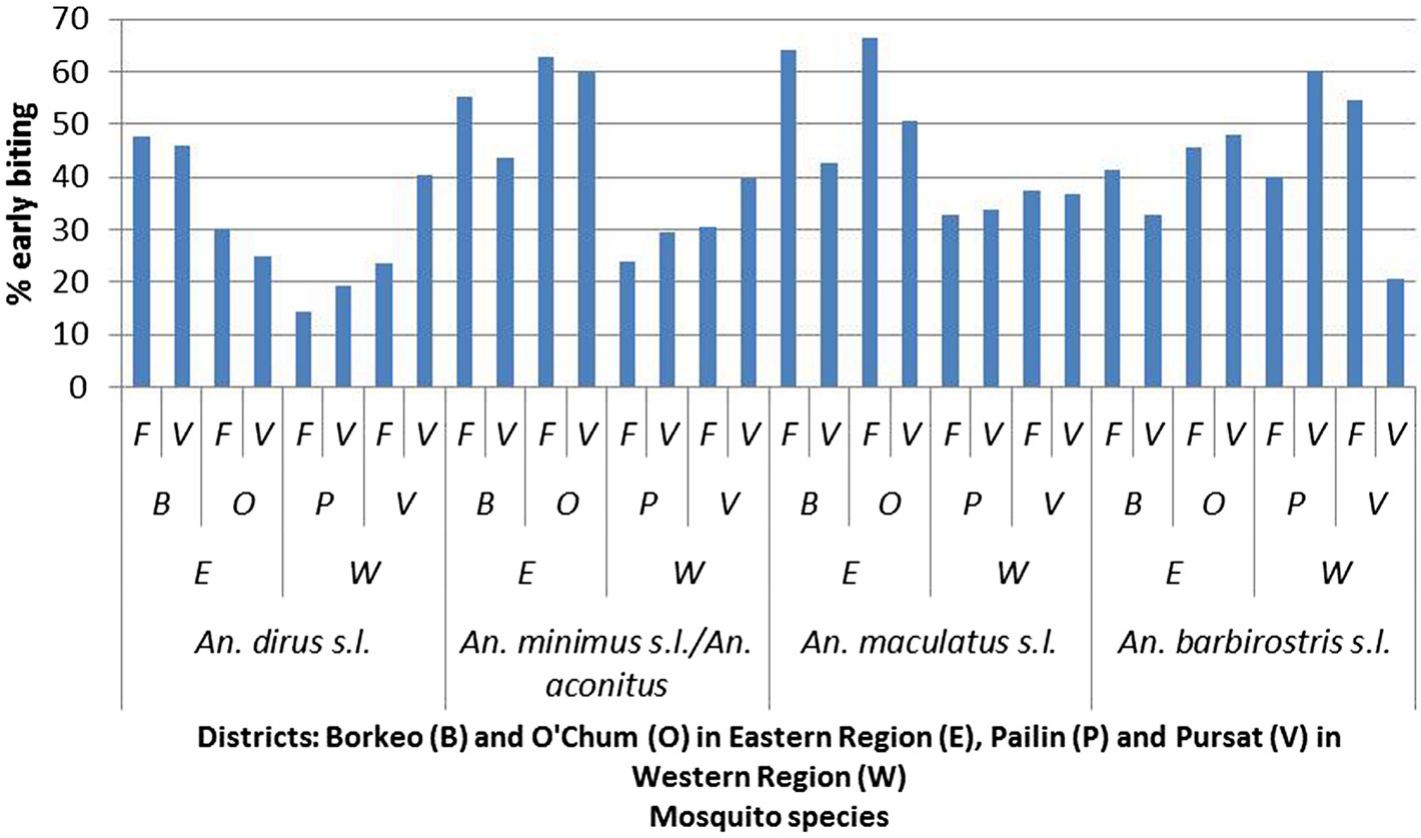
**Early biting rate for the different vector species complexes per collection site.** (**F** = forest, **V** = village), districts (**B** = Borkeo, **O** = O’Chum, **P** = Pailin, **V** = Pursat), and region (**E** = east, **W** = west).

The EBP, calculated as the percentage of vectors biting before 22.00, varied according to district and less according to site (forest or village) (Figure 4).

Analysis by CART shows that also for the EBP, village is the most important determinant (RI: 100 for all species). Region and districts are also important determinants, except for the EBP of *An*. *barbirostris s*.*l*. (Table 5).

**Table 5:** **Ranking of predictor variables of *Anopheles *early biting activity by their relative importance (RI) as discriminant**

		** RI per mosquito species complex**		
**Discriminants**	***An. dirus s.l.***	***An. minimus s. l. / An. aconitus***	***An. maculatus s.l.***	***An. barbirostris s.l.***
Village	100.00	100.00	100.00	100.00
District	73.79	86.51	80.84	1.01
Region (East/West)	55.48	86.51	79.52	0.66
Site (Forest/Village)	10.51	0.00	0.37	12.22
Survey	25.80	0.00	0.21	10.05

**Figure 5:**
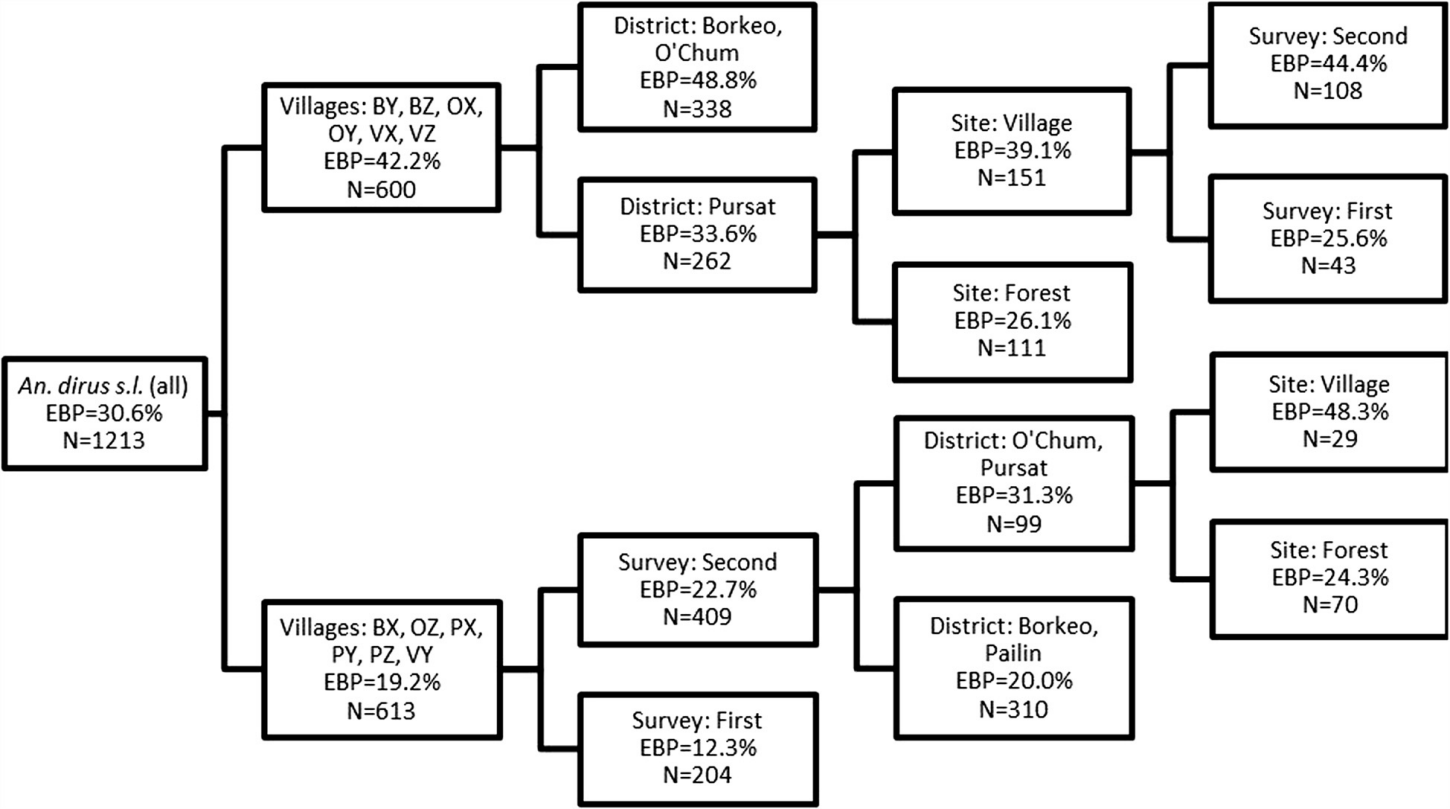
**Regression tree representing the important determinants for *Anopheles dirus s.l. *early biting proportion (EBP), expressed as percentage of vectors biting before 22.00.** The selected splitter variables (village, survey, district, site) are shown in the nodes.

For *An*. *dirus s*.*l*. (Figure 5), the highest EBP (49%) is observed in the districts of Borkeo (BY, BZ) and O’Chum (OX, OY). In two villages in Pursat (VX and VZ) a higher EBP was seen in the village (39%) as compared to the forest (26%). This was also observed in the second survey for OZ and VY (48% in the village *versus* 24% in the forest). The lowest EBP was seen in Pailin (Figure 4).

The CART trees for *An*. *minimus s*.*l*./*An*. *aconitus*, *An*. *maculatus s*.*l*. and *An*. *barbirostris s*.*l*. are shown in Additional file 2. For *An*. *minimus s*.*l*./*An*. *aconitus* and *An*. *maculatus s*.*l*., most villages with a higher EBP (54.4 and 56.6% for *An*. *minimus s*.*l*./*An*. *aconitus* and *An*. *maculatus s*.*l*. respectively) were located in Borkeo and O’Chum (east), whereas most villages with a lower EBP (26.9 and 32.9% for *An*. *minimus s*.*l*./*An*. *aconitus* and *An*. *maculatus s*.*l*. respectively) were located in Pailin and Pursat (west). For *An*. *barbirostris s*.*l*., in general an EBP of 45.8% was observed, with almost no difference between districts. A higher EBP (60.4%) was observed in four villages (OY, PY, PZ, VX) as compared to the others (38.3%).

### Malaria transmission

Some 10,080 specimens were tested for detection of sporozoites. The details of 9,233 of these specimens can be found in [21]. In addition to these, 826 *An*. *philippinensis* and 21 specimens of other anopheline species (11 *An*. *culicifacies s*.*l*., seven *An*. *hyrcanus*, two *An*. *aconitus* and one *An*. *tessellatus*) were tested. Only 17 specimens were confirmed to be positive for sporozoites (11 *P*. *falciparum*, one *Plasmodiummalariae*, five *P*. *vivax*). No mixed infections were observed. Only *An*. *dirus s*.*s*. was found positive for *P*. *falciparum* (11 specimens) and only during survey 1. Most of *P*. *falciparum* positives (9/11) were collected in the forest camps (BY and BZ). The five *P*. *vivax*- infected mosquitoes (four *An*. *dirus s*.*s*. and one *An*. *minimus s*.*s*.) were only found during survey 2, of which four in the villages. One specimen of *An*. *barbirostris s*.*s*. was found positive for *P*. *malariae* (forest camp of OZ).

**Figure 6:**
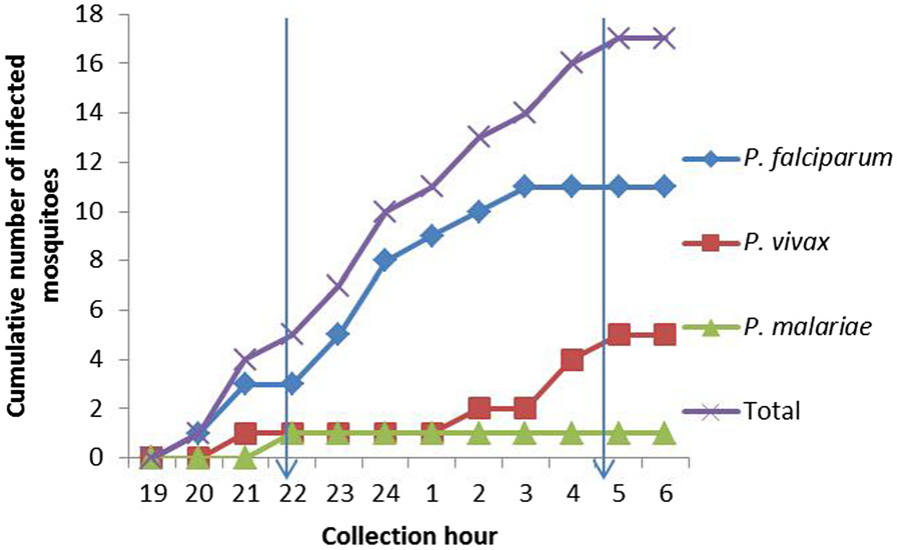
**Cumulative number of infected *Anopheles *mosquitoes (n = 17) during the night outdoors (all data pooled).** The approximate time for bed net use is between 22.00 and 05.00 (marked by arrows).

Based on all positive bites, the proportion of positive bites before sleeping time (22.00) was 29% (5/17) (Figure 6).

The EIR was calculated per week (Table 6). Malaria transmission was detectable in the four districts and not only in the forest, but also in the village sites. Transmission was very high in the forest camps of Borkeo with an average of 1.796 positive bites per week but with differences according to the collection place (3.422 in BY, 2.005 in BZ, 0 in BX).

**Table 6:** Entomological inoculation rate (EIR) per week

			**EIR/week**		
**District**	**Survey**	**Site**	***P. falciparum***	***P. vivax***	***P. malariae***
Borkeo	S1	Forest	1,796^1^		
		Village	0,302^1^		
	S2	Forest		0,159^1^	
Ochum	S1	Forest			0,225^3^
Pailin	S1	Village	0,214^1^		
	S2	Village		0,402^2^	
Pursat	S1	Village		0,219^1^	
	S2	Village		0,183^1^	

Only values different from zero are reported.

^1^ Only *An*. *dirus s*.*s*. contributes to this EIR.

^2^ Both *An*. *dirus s*.*s*. and *An*. *minimus s*.*s*. contribute to this EIR.

^3^ Only *An*. *barbirostris s*.*s*. contributes to this EIR.

### Relation between entomological and epidemiological findings

The results of the multivariate linear regression analyses are shown in Table 7, with detailed results of univariate and multivariate analyses in Additional file 3. The EIR is the only variable positively linked with the seroconversion rate or parasite rate: a higher *P*. *falciparum* EIR in Survey 1 is linked to a higher *P*. *falciparum* seroconversion rate in survey 2, and a higher *P*. *falciparum* parasite rate in survey 1. A similar trend was observed for the *P*. *vivax* EIR in survey 1, which was positively linked with the *P*. *vivax* seroconversion rate in survey 2. The densities and early biting rates of the different vector species were either not or negatively correlated to the seroconversion rate or parasite rate, showing that vector density is not a good proxy of transmission.

**Table 7:** Results of the multivariate regression analysis

**Independent variables**	**Dependent variable**	**Coefficient**	**P-value**
EIR PF S1	PF SCR S2	0,2492972	0,000
EIR PF S1	PF PR S1	0,1422721	0,022
EIR PV S1	PV SCR S2	1,224645	0,003
MBR barb S1	PV PR S2	−0,0224375	0,015
MBR dir S1	PF SCR S2	−0,0305142	0,025
MBR mac S2	PF SCR S2	−0,0116138	0,002
MBR mac S1	PF PR S2	−0,0094052	0,089
EBP min S1	PF SCR S1	−0,2482919	0,021
EBP min S1	PV PR S1	−0,3513601	0,040

*PFP*. *falciparum*, *PVP*. *vivax*, *SCR* seroconversion rate, *PR* parasite rate as detected by microscopy, *S1* survey 1; *S2* survey 2, *MBR* man biting rate, *EBP* early biting proportion, *EIR* weekly entomological inoculation rate, dir: *An*. *dirus s*.*l*.; min: *An*. *minimus s*.*l*.*/An. aconitus*; mac: *An*. *maculatus s*.*l*.; barb: *An. barbirostris s.l.*.

## Discussion

Vector control has always been one of the cornerstones of malaria control [27]. However, for vector control and the evaluation of its efficacy it is important to know the behaviour of the targeted vectors. As forest malaria is one of the major challenges in the elimination of malaria in Southeast Asia [7], the present study evaluated the vector density, early biting activity and malaria transmission of outdoor biting malaria vectors in two forested regions in Cambodia.

While all vector complexes occurred in all study villages, most of the variability in mosquito densities and early biting rate was explained by geographical factors, which was mainly at village level, and to a lesser extent at district and regional level. Cook *et al*. [19] also observed that malaria transmission as measured by serological markers can be linked to a certain village, and unpublished incidence data based on rapid diagnostic tests show a high within village correlation of the malaria incidence in 2010 and 2011 in Cambodia (Somony Heng, in preparation). This means that some villages are more prone to malaria transmission, while some are more prone to higher (or lower) vector densities, although, as discussed below, vector density does not linearly relate to malaria transmission. Variability in mosquito density also occurred between regions and surveys. In the east, higher *An*. *dirus s*.*l*. and *An*. *maculatus s*.*l*. densities were observed in survey 1 as compared to survey 2, whereas in the west, higher densities were observed in survey 2. However, in the east, the amount of rain is almost double compared to the west, with the main rain peak occurring in July and August. In the west, the rain peak occurs in September and October. This difference in rain pattern might explain these general differences observed in vector densities between both regions. The surveys were carried out at approximately the same moment in time in the east and the west. Therefore, survey 1 fell just after the rain peak in the east, and before the rain peak in the west (Figure 2), whereas survey 2 was at the start of the dry season in both regions. The survey effect on the mosquito densities should thus be interpreted with caution. For the *An*. *maculatus* complex this difference between east and west could also be related to a different proportion of *An*. *maculatus s*.*s*. versus *An*. *sawadwongporni*. However, a previous study in Thailand has shown that these species have a similar association to rainfall [28]. Despite the lower total amount of rain, the highest densities of *An*. *dirus s*.*l*., *An*. *minimus s*.*l*./*An*. *aconitus*, and *An*. *maculatus s*.*l*. were observed in villages in the west (Figure 3), showing that not only the amount of rain, but also other ecological factors are important determinants for mosquito density.

Strikingly, no difference was observed in vector densities between the village sites and the corresponding forest camps for *An*. *dirus s*.*l*., which is a forest-breeding species [7,29]. This was not only due to the close distance between villages and related forest plots, as in villages where the forest plots were further away (PX and VY), still comparable densities of malaria vectors were observed; in villages where the forest plot was very close (BY, BZ, OZ), higher densities of *An*. *dirus s*.*l*. were observed in the forest camps (7.08 BMN) as compared to the nearby villages (4.23 BMN), but only in the first survey. The latter three villages are situated in areas with the highest degree of conserved forest as compared to the other villages in the study (Table 1), confirming that higher degrees of conserved forest sustain higher *An*. *dirus s*.*l*. densities in the forest [29]. Nevertheless, present results contrast with previous studies reporting much higher *An*. *dirus s*.*l*. densities and malaria transmission in forest plots as compared to nearby villages, including a study in Pailin district [30] at a time when forest was still conserved, and studies in Central Vietnam (Khanh Hoa province [31], Ninh Thuan province [11]), and in south-western Vietnam (Binh Phuoc province [32]). In the early 2000s, the bed-net use in Vietnamese villages was higher [11] than the one observed during the current study performed in 2005 in Cambodia, which could explain the lower vector densities in the villages in Vietnam.

In the present study, in general, no difference was observed in the densities of the other vectors *An*. *minimus s*.*l*./*An*. *aconitus*, *An*. *barbirostris s*.*l*. and *An*. *maculatus s*.*l*. between forest plots and villages. These malaria vector complexes do not require the forest for breeding sites; *An*. *minimus s*.*l*. is more associated with mosaic vegetation and crop [9], and *An*. *maculatus s*.*l*. is a widespread species [9] preferring open or only partially shaded breeding sites, similar to *An*. *barbirostris s*.*l*. and *An*. *aconitus*[33].

Although the densities of the malaria vectors differed between villages, all vector complexes were found in all villages and forest plots, regardless if the villages were surrounded by deforested areas or by forest (evergreen or deciduous), or plantations. The forest in the region where this study took place was in most cases scattered and fragmented. It has been reported that *An*. *dirus s*.*l*. is able to adapt to peripheral areas where natural forests are replaced with orchards, and tea, coffee, and rubber plantations [29]. It is not yet clear however if these vectors have the ability to totally adapt to these new habitats in case of total deforestation. They probably still need patches of forest to survive during the dry season [7,29], from which they can spread to other breeding areas, such as plantations, in the wet season. The fact that in the present study these vectors remain present, even in very deforested areas, suggests that even a small patch of fragmented forest would be enough, or that *An*. *dirus s*.*l*. is able to overcome larger distances than the 1.5 up to 3 km in [29] commonly thought. Therefore vector control measures should focus on these important vectors [34], even in very deforested areas, and should take into account their early- and outdoor-biting behaviour. Moreover, areas with high forest fragmentation will be more accessible to people [5], meaning that the vector-host contact could also increase with increasing forest fragmentation.

Although densities were almost similar between villages and nearby forest plots, 11 (65%) out of 17 infective bites were found in the forest plots of BY, BZ and OZ, which are the villages with the highest degree of conserved forest, being in line with previous observations of forest-related transmission [11,29,35]. However, as shown by the current entomological data and the seroprevalence data [19], a considerable part of the transmission also occurs in the villages. This is in contrast to previous observations in Cambodia and Vietnam, where transmission is mostly limited to forest [11,31,35] and the way to the forest [11].

In the study regions, people combine living in the villages with a second house near to their farm plot in the forest. In 2005, a great proportion of people sleeping in the forest slept unprotected (17 to 31%) particularly in Borkeo, the district with the highest transmission rate. It is clear that both human behaviour (sleeping unprotected in the forest, outdoor activities in the early evening in the village, sleeping unprotected in the village) [36] and vector behaviour (biting outdoors and early) provide ample opportunity for malaria transmission.

The data from the current study show that LLINs provide useful although only partial protection against malaria: 71% of the infectious bites occurred after 22.00, when people are expected to be sleeping under a net. All infective bites before 22.00 (29%) occurred in the forest plots, underlining the importance of additional protective methods in such environments (eg long-lasting insecticidal hammocks [14], topical or spatial repellents). In the western part of the country malaria transmission, mainly *P*. *vivax*, occurred later in the night and was only observed inside the villages and not in the forest plot. It has been observed that the use of LLINs can alter the biting behaviour of the vector species to earlier biting [37], either through selective pressure of the LLINs resulting in, for example, species replacement, or through adaptation of the vector species through phenotypic plasticity [15]. In the Ninh Thuan province in Vietnam, where LLIN use was reported to be much higher (85% in the villages and 53% in the forest) than in the Cambodian study region in 2005, a higher proportion of vector bites occurred before sleeping time [11] (61% in the villages, and 45% in the forest plots *versus* 37 and 38%, respectively for the present study). Interestingly, in some villages located in O’Chum and Pursat districts, a higher proportion of early biting *An*. *dirus s*.*l*. was observed in the villages (39%) as compared to the forest plots (26%). In these districts, people reported a high use of bed nets (treated and untreated) in the village, but not in the forest. An increasing trend of early biting due to increasing LLIN use cannot be excluded. The current study took place in 2005, when LLIN coverage and use was much lower than presently observed. The Cambodia Malaria Survey shows a national increase in LLIN use from 29 and 25% in 2004 and 2007, respectively, until more than 50% in 2010, both in the east and the west [38]. This coverage has increased in 2011 and 2012 after massive distribution campaigns of LLINs. Therefore, the present survey could serve as a baseline for future studies on the effect of vector control measures in Cambodia.

Besides the primary vectors *An*. *dirus s*.*s*. and *An*. *mini-mus s*.*s*., other vectors can also transmit malaria. The current study is the first to find *An*. *barbirostris s*.*s*. positive for *P*. *malariae* in Cambodia. *An. barbirostris s*.*l*. is a confirmed vector of *P*. *falciparum* in Timor, based on salivary gland infection [39], and has been found positive in CSP-ELISA in Indonesia [40], Sri Lanka [41] and Thailand [42]. These ELISA results however were not confirmed by PCR or heating the ELISA lysates, and as such they might consist of false positive reactions as reported earlier [21]. In a malaria-endemic region in Thailand, *An*. *barbirostris s*.*l*. is highly suspected for maintaining malaria transmission in the absence of the main vectors [43], but, despite the observed high densities (up to 14 B/M/N in the O’Chum district), the importance of *An*. *barbirostris s*.*l*. as a secondary vector in Cambodia is not known. In Thailand [33] and in Vietnam [11,44], also members of the *An*. *maculatus* group have been found to carry *Plasmodium* sporozoites and are considered important primary or secondary vectors. In the current study however, none of more than 3,500 *An*. *maculatus s*.*l*. tested positive in the CSP-ELISA. Moreover, this study has only focused on the secondary vectors *An*. *barbirostris s*.*l*. and *An*. *maculatus s*.*l*.. Given the diversity of anophelines collected, more potential secondary vectors may be present, although not infected at the time of the surveys. Even if these secondary vector species on their own cannot maintain malaria transmission, co-occurrence of several secondary vector species could constitute a vector population which is capable of maintaining malaria transmission [45]. For example, in Vietnam, a combination of secondary vectors was shown to maintain transmission, though at a low rate [44].

Within the primary and secondary vectors, there is a large variation in anthropophily, exophily and early biting activity between regions in Southeast Asia [10,12]. In the current study it has been confirmed that, as in other parts of Southeast Asia [12,13,28] the studied vectors in Cambodia bite early and outdoors. The combination of domestic animals present in all study villages, and the zoophilic behaviour of these primary [12,46] and secondary [10,28] vectors, reduces the impact of vector control measures such as ITNs and indoor residual spraying and alternative vector control methods should be explored [47].

The results of the serological evaluations of antibody responses to *P*. *falciparum* and *P*. *vivax* in relation to risk factors is discussed in detail elsewhere [19]. The current study has focused on a possible relation between the entomological and parasitological or serological data only. This study clearly shows that vector densities are not, or even negatively, correlated with the seroconver-sion rate. Therefore vector densities cannot be used as proxy for malaria transmission. As expected, the strongest predictor of the *P*. *falciparum* and *P*. *vivax* seroconversion rate in survey 2 was the *P*. *falciparum* and *P*. *vivax* EIR of survey 1. Therefore, this study confirms previous findings of correlation between entomological transmission and serological surveys [18]. Serological markers of transmission show greater sensitivity in low transmission areas, as seroprevalence reflects cumulative exposure and thus is less affected by seasonality due to the longer duration of specific antibody responses [17,18]. However an entomological survey using human landing as the collection method is the only way to identify vector species involved in malaria transmission and their biting behaviour. As in the present study, the analysis was carried out on village level, it shows that the EIR is still an important tool in documenting trends in malaria transmission at local level, even in areas with low malaria transmission intensity, such as Cambodia.

## Conclusions

The data presented in the current study, based on outdoor human landing collections, clearly emphasizes the importance of outdoor malaria transmission in the forest as well as in the village. Although vector species are present in all sampled forested villages (12), their densities vary according to villages, rather than region, without major differences between the sites of collections (inside the village or in the nearby forest plot). The consequences of forest fragmentation and deforestation on malaria transmission in Southeast Asia are difficult to predict as a wide diversity of forest, near-forest and non-forest malaria vectors occurred. A suboptimal habitat in the forest fragments might result in lower densities and survival of the main forest vectors, with a reduction of malaria transmission as a consequence. However, higher accessibility of forest fragments can result in a higher contact rate between man and vector. Additionally, in the long run, deforested areas or areas with fragmented forest might be invaded by other efficient malaria vectors which are now considered secondary vectors. Therefore, although the capacity of secondary vectors of Cambodia in maintaining malaria transmission in the absence of the primary vectors is not known, their behaviour should also be taken into account when applying vector control measures. Because of outdoor and early biting by primary and secondary vectors in Cambodia, a behavioural trait that can be selected in vectors by a higher use of LLINs, additional measures should be explored. Personal protection using LLIHs, or topical and spatial repellents can have added value in tackling residual malaria transmission.

## Competing interests

The authors declare that they have no competing interests.

## Author’s contributions

MC and TS designed the study. LDu and MC carried out the data analysis and drafted the manuscript. TS and SM facilitated and supervised the field work, and critically reviewed the manuscript. LDe and PR carried out the ELISA assays and molecular identification of the collected mosquitoes and critically reviewed the manuscript. MC critically reviewed the manuscript. All authors read and approved the final manuscript.

## Supplementary Material

Additional file 1 **Regression trees for densities of *Anopheles dirus s.l., Anopheles minimus s.l./Anopheles aconitus, Anopheles maculatus s.l., *and *Anopheles barbirostris s.l. ***The data provided represent the result of the CART analysis for man biting rates of *Anopheles dirus s*.*l*., *Anopheles minimus s*.*l*./*Anopheles aconitus*, *Anopheles maculatus s*.*l*., and *Anopheles barbirostris s*.*l*. Click here for file

Additional file 2 **Regression trees for early biting rates of *Anopheles minimus s.l./Anopheles aconitus, Anopheles maculatus s.l. *and *Anopheles barbirostris s.l. ***The data provided represent the result of the CART analysis for early biting rates of *Anopheles minimus s*.*l*./*Anopheles aconitus*, *Anopheles maculatus s*.*l*., and *Anopheles barbirostris s*.*l*. Click here for file

Additional file 3 **Results of univariate and multivariate analysis for seroconversion rate and parasite rate.** This table shows the coefficients and the p-values of the univariate and multivariate regression analysis for each of the dependent and independent variables assessed. Click here for file
