# Supplementary material for: Outdoor malaria transmission in forested villages of Cambodia
Source: Malar J. 2013 Sep 17;12:329. doi: 10.1186/1475-2875-12-329 (PMC3848552; doi:10.1186/1475-2875-12-329)
Supplement: Additional file 1 — Regression trees for densities of Anopheles dirus s.l., Anopheles minimus s.l./Anopheles aconitus, Anopheles maculatus s.l., and Anopheles barbirostris s.l. The data provided represent the result of the CART analysis for man biting rates of Anopheles dirus s.l., Anopheles minimus s.l./Anopheles aconitus, Anopheles maculatus s.l., and Anopheles barbirostris s.l. [file 1475-2875-12-329-S1.docx]

**Additional File 1: Regression trees for densities of *An. dirus s.l., An. minimus s.l./An. aconitus, An. maculatus* *s.l.,* and *An. barbirostris s.l.***

Additional Figure 1. 1: Regression tree representing the important determinants for *An. dirus s.l.* density (MBR=man biting rate). The selected splitter variables (village, survey, site) are shown in the nodes. SD=Standard Deviation; N=Number of man-collection nights on which the data are based

Additional Figure 1. 2: Regression tree representing the important determinants for *An. minimus s.l./An. aconitus* density. The selected splitter variables (villages, survey) are shown in the nodes. SD=Standard Deviation; N=Number of man-collection nights on which the data are based.

Additional Figure 1. 3: Regression tree representing the important determinants for *An. maculatus s.l.* density. Village is the only selected splitter variables. SD=Standard Deviation; N=Number of man-collection nights on which the data are based.

Additional Figure 1. 4: Regression tree representing the important determinants for *An. barbirostris s.l.* density. Village and collection site are the selected splitter variables, shown in the nodes. SD=Standard Deviation; N=Number of man-collection nights on which the data are based.
