# Supplementary material for: Outdoor malaria transmission in forested villages of Cambodia
Source: Malar J. 2013 Sep 17;12:329. doi: 10.1186/1475-2875-12-329 (PMC3848552; doi:10.1186/1475-2875-12-329)
Supplement: Additional file 2 — Regression trees for early biting rates of Anopheles minimus s.l./Anopheles aconitus, Anopheles maculatus s.l. and Anopheles barbirostris s.l. The data provided represent the result of the CART analysis for early biting rates of Anopheles minimus s.l./Anopheles aconitus, Anopheles maculatus s.l., and Anopheles barbirostris s.l. [file 1475-2875-12-329-S2.docx]

**Additional File 2: Regression trees for early biting rates of *An. minimus* *s.l./An. aconitus, An. maculatus* *s.l.,* and *An. barbirostris s.l.***

Additional Figure 2. 1: Regression tree representing the important determinants for *An. minimus s.l.*/*An. aconitus* early biting rate. Village is the only selected splitter variable.

Additional Figure 2. 2: Regression tree representing the important determinants for *An. maculatus* *s.l.* early biting rate. The selected splitter variables (village, site and district) are shown in the nodes.

Additional Figure 2. 3: Regression tree representing the important determinants for *An. barbirostris s.l.* early biting rate. The selected splitter variables (village, survey and site) are shown in the nodes.
