# Supplementary material for: Outdoor malaria transmission in forested villages of Cambodia
Source: Malar J. 2013 Sep 17;12:329. doi: 10.1186/1475-2875-12-329 (PMC3848552; doi:10.1186/1475-2875-12-329)
Supplement: Additional file 3 — Results of univariate and multivariate analysis for seroconversion rate and parasite rate. This table shows the coefficients and the p-values of the univariate and multivariate regression analysis for each of the dependent and independent variables assessed. [file 1475-2875-12-329-S3.docx]

**Additional File 3: Results of univariate and multivariate analysis for seroconversion rate and parasite rate**

| Dependent variable | Analysis | Independent variables | Coefficient | P-value |
| --- | --- | --- | --- | --- |
| PF SCR S1 | univariate | EBP min S1 | -0,2482919 | 0,021 |
| PF SCR S2 | univariate | MBR dir S1 | 0,0353274 | 0,054 |
|  |  | EBP dir S1 | 0,5866511 | 0,015 |
|  |  | EIR PF S1 | 0,1827394 | 0,001 |
|  |  | MBR dir S2 | -0,032255 | 0,063 |
|  |  | MBR mac S2 | -0,0133728 | 0,063 |
|  | multivariate | MBR dir S1 | -0,0305142 | 0,025 |
|  |  | EIR PF S1 | 0,2492972 | 0,000 |
|  |  | MBR mac S2 | -0,0116138 | 0,002 |
|  |  | constant | 0,2360907 | 0,000 |
| PF PR S1 | univariate | EIR PF S1 | 0,1422721 | 0,022 |
|  |  | EBP mac S1 | 0,610567 | 0,075 |
|  | multivariate | EIR PF S1 | 0,1422721 | 0,022 |
|  |  | constant | 0,1303814 | 0,010 |
| PF PR S2 | univariate | MBR mac S1 | -0,0094052 | 0,089 |
| PV SCR S1 | univariate | MBR min S1 | 0,0124118 | 0,087 |
|  |  | EBP dir S1 | -0,2784758 | 0,074 |
|  |  | EBP min S1 | -0,2793448 | 0,068 |
|  | multivariate | constant | 0,1640838 | 0,001 |
| PV SCR S2 | univariate | EIR PV S1 | 1,204488 | 0,001 |
|  |  | MBR dir S2 | 0,0253302 | 0,065 |
|  |  | EBP bar S1 | -0,2724033 | 0,07 |
|  | multivariate | EIR PV S1 | 1,224645 | 0,003 |
|  |  | constant | 0,1204187 | 0,003 |
| PV PR S1 | univariate | MBR bar S1 | -0,0182514 | 0,046 |
|  |  | MBR min S1 | 0,0211289 | 0,058 |
|  |  | EBP min S1 | -0,3513601 | 0,04 |
|  |  | EBP dir S1 | -0,3892562 | 0,094 |
|  | multivariate | EBP min S1 | -0,3513601 | 0,040 |
|  |  | constant | 0,4003114 | 0,000 |
| PV PR S2 | univariate | MBR bar S1 | -0,0224375 | 0,015 |
|  |  | EBP dir S2 | -0,4472159 | 0,023 |
|  |  | EBP mac S2 | -0,3838485 | 0,028 |
|  |  | EBP min S2 | -0,5776682 | 0,023 |
|  |  | MBR dir S2 | 0,031092 | 0,056 |
|  | multivariate | MBR bar S1 | -0,0224375 | 0,015 |
|  |  | constant | 0,2611861 | 0,000 |

PF: *P. falciparum*; PV: *P. vivax*; SCR: Seroconversion Rate; PR: Parasite rate as detected by microscopy; S1: Survey 1; S2: Survey 2; MBR: man biting rate; EBP: early biting proportion; EIR: weekly entomological inoculation rate; dir: *An. dirus* *s.l.*; min: *An. minimus* *s.l.*/*An. aconitus*; mac: *An. maculatus* *s.l.*; min: *An. minimus s.l.*
